# Applications of the Cellular Thermal Shift Assay to Drug Discovery in Natural Products: A Review

**DOI:** 10.3390/ijms26093940

**Published:** 2025-04-22

**Authors:** Jayoung Song

**Affiliations:** Department of Pharmacology, College of Medicine, Dankook University, Cheonan 31116, Republic of Korea; jsong@dankook.ac.kr; Tel.: +82-41-550-3874

**Keywords:** cellular thermal shift assay (CETSA), natural products, drug discovery, target identification, chemical biology

## Abstract

Natural products play a crucial role in drug discovery because of their structural diversity and biological activity. However, identifying their molecular targets remains a challenge. Traditional target identification approaches such as affinity-based protein profiling and activity-based protein profiling are limited by the need for chemical modification or reactive groups in natural products. The emergence of label-free techniques offers a powerful alternative for studying drug–target engagement in a physiological context. In particular, the cellular thermal shift assay (CETSA) exploits ligand-induced protein stabilization—a phenomenon where ligand binding enhances a protein’s thermal stability by reducing conformational flexibility—to assess drug binding without requiring chemical modifications. CETSA’s integration with advanced mass spectrometry and high-throughput platforms has dramatically expanded proteome coverage and sensitivity, enabling the simultaneous quantification of thousands of proteins and the identification of low-abundance targets in native cellular environments. This review highlights the application of key CETSA-based methods to target identification in natural products including Western blot-based CETSA, isothermal dose–response CETSA, mass spectrometry-based CETSA, and high-throughput CETSA. Case studies are presented that demonstrate their effectiveness in uncovering the mechanisms of action of different drugs. The current limitations of CETSA-based strategies are also explored, and future improvements to optimize their potential for drug discovery are discussed. Integrating CETSA with complementary approaches can enhance the target identification accuracy and efficiency for natural products and ultimately advance development of therapeutic applications.

## 1. Introduction

Natural products are an important resource in the field of drug discovery and development owing to their structural diversity and biological activity. Over the past 30 years, natural products have yielded numerous therapeutic agents for treating various diseases. From 1981 to 2019, 49.2% of all drugs approved by the Food and Drug Administration (FDA) were derived from natural products, including 63.2% of anticancer drugs [1]. The process of drug discovery and development typically begins with the isolation of bioactive compounds in natural products followed by the identification of their molecular targets, which is critical for understanding their mechanisms of action and potential side effects. Target identification has traditionally relied on phenotypic screening followed by biochemical assays, where the biological effects of the bioactive compounds on living systems are observed, such as phenotypic alterations and changes to related cellular signaling pathways. Various techniques are then used to identify the molecular targets responsible for these effects, among which labeling methods based on chemical proteomics are primarily used. These methods can be divided into two main categories: affinity-based protein profiling (AfBPP) and activity-based protein profiling (ABPP) [2]. AfBPP involves the transient immobilization of a natural product by using a tag such as biotin. The tag is inserted at a site that does not affect biological activity to facilitate immobilization after which the target proteins that interact with the natural product are extracted from the cell lysate. AfBPP has proven very effective and has been used to successfully identify the targets of many natural products such as Diazonamide A, FK506, Trapoxin, and Aurilide [3,4,5,6]. ABPP involves forming a covalent bond between the natural product and its target protein and thus requires the natural product to contain a reactive group that reacts with the target protein. Fortunately, many natural products do contain reactive groups such as epoxides, disulfides, lactones, and quinones, which has facilitated the wide application of this method [7]. However, both methods have notable limitations. AfBPP requires modifying a molecule to introduce an affinity tag, which may affect the biological activity, and it comes with the risk of nonspecific binding during the process of affinity purification. Meanwhile, ABPP is restricted to natural products that inherently possess reactive groups.

To overcome these limitations, label-free target identification methods have recently emerged as promising alternatives including the cellular thermal shift assay (CETSA), drug affinity responsive target stability (DARTS), and stability of proteins from rates of oxidation (SPROX). These methods eliminate the need for chemical modification of the natural product, which preserves its biological activity and enables more robust target identification. These methods instead detect changes in protein stability or other biophysical properties upon drug binding and thus provide an unbiased and efficient approach to identifying protein targets, particularly in natural products with complex structures that are challenging to modify chemically.

Table 1 below provides a comparative evaluation of CETSA, DARTS, SPROX, and traditional affinity-based methods. CETSA is particularly effective for studying kinases and membrane proteins in intact cells, making it ideal for assessing drug–target engagement under physiological conditions. SPROX excels in analyzing high molecular weight proteins, ATPases, and weak binders by detecting domain-level stability shifts, while DARTS/SPROX are optimal for label-free novel target discovery in lysates.

This review presents a comprehensive overview of CETSA and its advanced derivatives with a focus on their principles, methodologies, and applications in drug–target engagement studies. Representative case studies are presented to demonstrate how these techniques have facilitated target identification in natural products in cellular and proteome-wide contexts. Practical limitations and future improvements are also discussed for the wider application of CETSA-based strategies to target identification and drug discovery and development.

## 2. Overview

**Figure 1:**
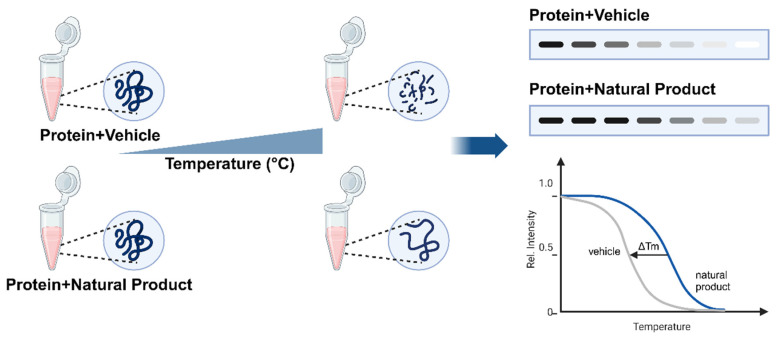
Principle of the cellular thermal shift assay (CETSA). When exposed to increasing temperature, proteins treated with a vehicle (control) undergo thermal denaturation and aggregation, which reduces their solubility. In contrast, proteins bound to a natural product exhibit enhanced thermal stability, and they maintain their solubility at higher temperatures. The remaining soluble protein fraction is analyzed via a technique such as Western blotting, where an increased band intensity at higher temperatures indicates protein stabilization by the natural product. Rel. Intensity: relative intensity, Tm: melting temperature.

First introduced in 2013, CETSA is a label-free biophysical technique of detecting drug–target engagement based on the ligand-induced thermal stabilization of proteins. A ligand that binds to a target protein enhances its thermal stability, which reduces its susceptibility to denaturation under thermal stress. Unlike traditional chemical proteomics that often require the chemical modification of compounds, CETSA directly assesses the changes in thermal stability of target proteins when they bind with small molecules, which provides a straightforward and physiologically relevant approach for studying drug–target engagement [10]. As shown in Figure 1, prepared samples are treated with a drug or control vehicle for a specified time and are then subjected to a temperature gradient. Proteins bound to the drug remain stable while the unbound proteins denature and precipitate with increasing temperature. Cells are then lysed through multiple freeze–thaw cycles (e.g., rapid freezing in liquid nitrogen followed by thawing at 37 °C), and the soluble protein fraction is separated from the aggregate by centrifugation or filtration. The thermally stabilized proteins are quantified by techniques such as Western blotting or mass spectrometry to generate thermal melting curves that indicate the protein melting point (Tm), and a shift in Tm (∆Tm) serves as a marker of drug–target engagement [11]. It is important to recognize that such a shift reflects the presence of drug–target engagement at the tested drug concentration, but it does not provide information about the degree of target binding or occupancy, which reflects the potency of the drug. The potency of a drug can be determined by isothermal dose–response CETSA (ITDR-CETSA), which involves applying a range of drug concentrations at a fixed temperature close to Tm of the unbound protein. Compounds are then ranked by their binding capacity based on the half-maximal effective concentration (EC_50_) [12].

## 3. CETSA-Based Strategies and Their Applications

### 3.1. Target Binding

**Figure 2:**
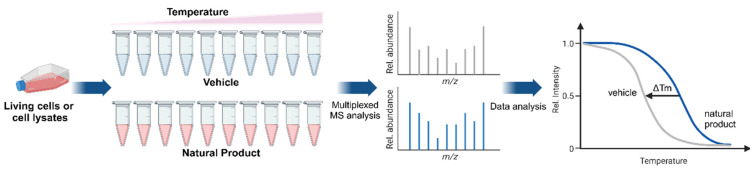
Workflow of MS-CETSA for target identification in natural products. Living cells or cell lysates are treated with either a vehicle or natural product and are subjected to a temperature gradient. The obtained soluble proteome is subjected to multiplexed mass spectrometry for proteome-wide analysis. Thermal stability curves are then generated, where a shift in the melting temperature (∆Tm) indicates drug–target binding and stabilization. MS analysis: Mass Spectrometry analysis, Rel. Intensity: relative intensity, Tm: melting temperature.

Western blotting CETSA (WB-CTSA) relies on Western blotting to detect specific proteins. It is a simple and label-free technique with the advantage of easy implementation in a standard laboratory without requiring specialized equipment. However, it has a limited throughput because it requires specific antibodies, so it is best suited for hypothesis-driven studies. Consequently, WB-CETSA is commonly employed to validate known target proteins rather than discover novel target proteins [10,13]. As noted earlier, ITDR-CETSA is used to measure the dose-dependent thermal stabilization of proteins at a fixed temperature, which allows for quantitative assessment of the drug-binding affinity and makes it a useful tool for ranking compounds targeting specific proteins [12]. As shown in Figure 2, mass spectrometry CETSA (MS-CETSA) is an advanced variant of CETSA that replaces Western blotting with mass spectrometry to detect changes in thermal stability across thousands of proteins simultaneously and provide comprehensive insights into drug-binding events. Thermal proteome profiling (TPP), which is often considered synonymous with MS-CETSA, extends this capability by incorporating gradients of temperature and compound concentrations to generate detailed thermal stability profiles. These profiles can then be used to quantitatively assess the drug-binding affinities and mechanisms of action with a high throughput [14]. These techniques are particularly powerful for drug discovery and mechanistic studies because they allow for the large-scale identification of drug targets and study of complex protein interaction networks in physiologically relevant contexts. However, implementing MS-CETSA or TPP is resource-intensive and requires advanced setups, complex data processing tools, and technical expertise. Despite these challenges, their ability to analyze the entire proteome in an unbiased manner makes MS-CETSA and TPP invaluable tools for modern pharmacological research [15].

Two-dimensional TPP (2D-TPP) combines two experimental approaches to provide a more comprehensive and multidimensional understanding of drug–protein interactions: TPP based on temperature range (TPP-TR) and TPP based on compound concentration range (TPP-CCR). TPP-TR profiles changes in thermal stability of proteins across a temperature range, which makes it conceptually similar to classic CETSA and allows for the identification of temperature-dependent drug–protein interactions. TPP-CCR examines changes in protein stability across a gradient of compound concentrations, which is similar to ITDR-CETSA and helps quantify and rank the drug-binding affinities of compounds based on their potency [11,14]. Thus, 2D-TPP integrates the principles of TPP-TR and TPP-CR to analyze the thermal stability of proteins for two variables (i.e., temperature and compound concentration) simultaneously. This synergistic framework provides a high-resolution view into both the binding dynamics and multidimensional behavior of ligand–target engagement and makes 2D-TPP a powerful tool for advancing drug discovery [11]. Other notable CETSA variants have been developed to enhance the flexibility and applicability of the technique. Thermal stability shift-based fluorescence difference in two-dimensional gel electrophoresis (TS-FITGE) combines thermal stability analysis with fluorescence-based two-dimensional gel electrophoresis to identify drug-induced changes in protein stability in complex biological samples at a high resolution. Simplified TTP (STPP) reduces the complexity of the TPP workflow to improve throughput and accessibility without compromising the ability to detect critical protein–ligand interactions. These variants complement the broader CETSA family by addressing specific experimental needs and facilitating the study of drug–protein interactions in diverse biological contexts [15].

Other label-free target identification techniques such as DARTS and SPROX have often been applied with CETSA in a complementary manner to strengthen target validation. DARTS is based on the observation that binding with a ligand often stabilizes proteins against proteolytic degradation. Thus, target proteins can be identified by applying limited proteolysis to a protein mixture with and without a ligand and finding proteins that show increased resistance to proteases. SPROX measures ligand-induced changes in protein stability by tracking methionine oxidation rates as the concentration of a chemical denaturant is increased. Both methods complement CETSA by probing ligand-induced conformational and stability changes through distinct biophysical readouts, which expands the toolkit for drug–target discovery [16].

### 3.2. Drug–Target Engagement

CETSA and its variants are widely utilized to provide critical insights into the mechanisms of action and therapeutic potential of natural products. WB-CETSA was used to confirm that curcumol binds to nucleolin, matrine binds to Src, and rapanone A binds to IMPDH2, which helped identify their respective anticancer and anti-inflammatory effects [17,18,19]. WB-CETSA was also used to identify the engagement between Class III PI3K (Vps34) and an aurone derivative and the direct binding of 2′-hydroxycinnamaldehyde to STAT3, which stabilized the target proteins and inhibited their activity [20,21]. ITDR-CETSA was employed to study the interaction of oridonin with nucleolin, where a series of experiments including time-course and dose–response assays helped identify its EC50 value and optimal treatment conditions [22]. These examples demonstrate the versatility of WB-CETSA and ITDR-CETSA in validating interactions between natural products and target proteins in diverse biological systems.

MS-CETSA and TPP have been widely used for proteome-wide target identification, which enables the study of drug–protein interactions in a high-throughput manner. MS-CETSA was used to investigate the binding spectrum of staurosporine, which was a pan-kinase inhibitor derived from *Streptomyces staurosporeus*. In this study, thermal stability shifts were observed in 51 of the 175 kinase targets tested, which demonstrated its broad inhibitory activity [14]. MS-CETSA has also been used to study natural products with poorly understood mechanisms of action. For instance, MS-CETSA was used to identify the target proteins of quinine, which is an antimalarial drug derived from the bark of the cinchona tree. Purine nucleoside phosphorylase (PfPNP) was identified as a critical target, which helped elucidate the mechanism of action of quinine. This finding was further supported by biophysical and structural studies that confirmed that ligand binding induces conformational changes in PfPNP and helped clarify its antimalarial properties [23]. In another study, MS-CETSA was used to investigate the molecular target of iopromide A (VioA), which is a potent anticancer natural product derived from *Cystobacter violaceus*. While traditional affinity-based methods failed to identify a clear target, MS-CETSA revealed that VioA stabilized six proteins including nucleolar protein 14 (NOP14). Further studies demonstrated that VioA disrupted ribosome biogenesis by impairing the interaction between NOP14 and NEP1, which ultimately interfered with cancer cell proliferation [24]. MS-CETSA was also applied to study the targets of atractylenolide I, which is a bioactive natural product from *Atractylodes macrocephala*. Proteome-wide TPP-TR of colorectal tumor cells identified the proteasome 26S subunit non-ATPase 4 (PSMD4) as a key target of atractylenolide I. Binding to PSMD4 enhanced immunoproteasome activity, increased antigen presentation, and improved the immune response to cancer cells [25]. MS-CETSA was also used to show that alantolactone, which is a sesquiterpene lactone from *Inula helenium*, binds directly to AKR1C1 in NCI-H460 cells, which established its potential as a potent inhibitor of non-small-cell lung cancer [26]. MS-CETSA was also used to reveal that ainsliadimer A targets PRDX1 and PRDX2, which induces reactive oxygen species (ROS)-mediated apoptosis in colorectal cancer cells [27]. These examples demonstrate the effectiveness of MS-CETSA for the target identification of natural products.

These examples collectively demonstrate that CETSA is a highly valuable tool for uncovering the molecular targets of natural products with anticancer potential. Its ability to operate in physiologically relevant settings and detect ligand-induced thermal stabilization makes it particularly suited for cancer research, where target validation in complex biological systems is crucial. Recent studies further support this utility. For example, CETSA was used to show that capsaicin inhibits the G1 cyclin/CDK complex via direct binding to tNOX (ENOX2), resulting in G1 arrest in cancer cells [28]. Beyond this, CETSA has been instrumental in elucidating the mechanisms of established chemotherapeutic agents. In the case of 5-fluorouracil (5-FU), proteome-wide CETSA revealed not only its canonical inhibition of thymidylate synthase but also its destabilizing effects on RNA modification proteins such as ADAR and DKC1 in colorectal cancer cells, providing insights into its activity in drug-resistant models [29]. Together, these findings underscore CETSA’s broad applicability in anticancer drug discovery, especially for validating target engagement of structurally diverse and bioactive natural compounds.

As discussed above, representative examples of CETSA applications in natural product research are systematically summarized in Table 2, which highlights the natural products, their molecular targets, the study objectives, and the key findings.

### 3.3. Comparative Analysis of CETSA Applications: Natural Products vs. Synthetic Compounds

While CETSA has demonstrated broad utility in target identification for both natural products and synthetic compounds, distinct challenges and opportunities arise depending on the chemical nature of the molecule. Natural products often possess greater structural complexity, including higher molecular weight, multiple chiral centers, and diverse functional groups, which can enhance specificity but also complicate solubility and cell permeability. These characteristics may affect thermal stability shifts, making natural products more challenging to analyze via CETSA compared to synthetic molecules with more predictable behavior [8,30]. For example, sesquiterpene lactones like alantolactone require organic co-solvents (ex. DMSO) for solubilization, potentially altering the assay conditions [26]. In contrast, synthetic kinase inhibitors such as Palbociclib are optimized for aqueous compatibility, simplifying CETSA workflows [31].

Additionally, natural products frequently exhibit multi-target engagement, which may dilute observable ∆Tm values or obscure specific binding signals. Ainsliadimer A, a sesquiterpene dimer, simultaneously stabilizes PRDX1/2 and modulates NF-κB pathways, necessitating orthogonal validation (ex. DARTS, SPROX) to deconvolute polypharmacology [27]. Conversely, synthetic compounds like vemurafenib are designed for single-target specificity, yielding distinct thermal shifts that correlate with biochemical potency [12].

Therefore, when applying CETSA to natural products, challenges such as poor aqueous solubility, limited bioavailability, and chemical instability require tailored optimizations to ensure reliable target engagement detection. For example, prolonged incubation times (ex. 24 h for oridonin) may compensate for slow cellular uptake of high-molecular-weight compounds [22]. Temperature gradients and heating protocols are also adjusted to accommodate natural product-specific properties. For heat-labile compounds, shorter heating durations (ex. 3 min holds at 56 °C) minimize degradation while retaining target stabilization signals [32].

### 3.4. Integration of Multiple Techniques

Lv et al.’s study on ainsliadimer A (AIN), which identified PRDX1 and PRDX2 as targets leading to ROS-mediated apoptosis in colorectal cancer cells, serves as a representative example of the power of combining multiple techniques [27]. In this case, they integrated the complementary strengths of DARTS, TPP-TR, and WB-CETSA to comprehensively elucidate the molecular targets and mechanism of action of AIN, which is a sesquiterpene lactone dimer isolated from *Ainsliaea macrocephala*. AIN was initially suggested to exert its anticancer effects by inhibiting the NF-κB signaling pathway by targeting IKKα/β. However, because natural products commonly engage multiple targets, they employed a multiomics-based strategy to explore other potential targets. The results of connectivity map (CMAP) analysis, which is a transcriptome-based approach that compares gene expression signatures to identify potential drug–target relationships, indicated that PRDX1 may be a key target owing to the similarity between the gene expression signatures of AIN and withaferin A, which is a known PRDX1 inhibitor. They then employed DARTS to test this prediction, and several candidate binding proteins were identified including PRDX1 and PRDX2, which suggested that AIN may interact directly with these antioxidant enzymes. The results of TPP-TR also indicated interaction between AIN and PRDX1 and PRDX2. Following these initial findings, WB-CETSA was employed to confirm that AIN binds to PRDX1 and PRDX2 in a cellular context. The results provided direct biochemical evidence that AIN increases the thermal stability of PRDX1 and PRDX2 in intact colorectal cancer cells, which confirmed their role as cellular targets. Additional validation through site-directed mutagenesis and enzymatic assays revealed that AIN covalently binds to the critical cysteine residues of PRDX1 (i.e., Cys173) and PRDX2 (i.e., Cys172), which inhibits their peroxidase activity. They concluded that AIN induces oxidative stress and mitochondrial dysfunction by inhibiting PRDX1 and PRDX2, which results in ROS-mediated apoptosis in colorectal cancer cells. This study underscores how a multifaceted approach utilizing multiple orthogonal label-free strategies can facilitate comprehensive target identification and understanding of the mechanisms of action of natural products.

### 3.5. Focus on High-Throughput CETSA

**Figure 3:**
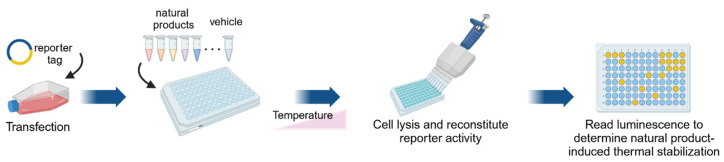
Workflow of the HT-CETSA reporter assay. Cells are transfected with a target protein fused to small reporter tags (e.g., ePL, 86b, HiBiT) and plated into a microtiter plate. After incubation with natural products and heat treatment, cells are lysed. Reporter activity is reconstituted by adding a large reporter fragment and substrate. Luminescence is then measured to assess protein stabilization using different complementation systems (ePL-EA complementation for β-galactosidase activity, 86b-11S and HiBiTLgBiT complementation for nanoluciferase activity).

High-throughput CETSA (HT-CETSA) was developed to facilitate the rapid and large-scale screening of compound libraries and identify drug leads that engage specific protein targets. HT-CETSA can be divided into two major approaches: HT-CETSA immunoassays and HT-CETSA reporter-based systems. HT-CETSA immunoassays evaluate the thermal stability of endogenous proteins by using antibody-based detection systems and frequently employ homogeneous detection platforms such as AlphaLISA/AlphaScreen, where proximity-based energy transfer between donor and acceptor beads occurs when an antibody binds to the target protein. Ligand-induced stabilization results in a stronger signal, which facilitates the quantification of target engagement in a high-throughput manner [13]. A HT-CETSA immunoassay was applied to assess B-Raf target engagement for 896 kinase inhibitors. Thirteen compounds were found to thermally stabilize B-Raf, which all had prior evidence of B-Raf binding [33]. Another study used a HT-CETSA immunoassay to screen a library of 10,928 compounds targeting thymidylate synthase, which identified 65 hits that stabilized the protein of which the majority were pyrimidine nucleoside analogs. Upon retesting the most potent stabilizers (>30% stabilization), 90% of the compounds were confirmed, which demonstrated the reliability of the HT-CETSA immunoassay for identifying novel ligands [29]. As shown in Figure 3, HT-CETSA reporter systems fuse a target protein with a reporter such as NanoLuciferase (NanoLuc) or β-galactosidase, and thermal stability changes are detected by luminescence or colorimetry. These fusion proteins can be introduced into cells through the transfection of genetic fusion construct or transient expression systems. Alternatively, they can be endogenously inserted into the genome using regularly clustered interspaced short palindromic repeats (CRISPR)-based techniques. All of these approaches can be used to monitor ligand-induced protein stabilization in live cells [34,35,36].

A NanoLuc-based live-cell thermal shift assay (NaLTSA) was developed to monitor the stabilization of kinases, bromodomains, and histone deacetylase upon ligand binding and was applied to assessing the stabilization of p38α kinase across a set of 80 kinase inhibitors. NaLTSA was also applied to profile the target engagement of ponatinib, which is a multikinase inhibitor, across 38 NanoLuc-tagged kinases. Out of 20 known targets of ponatinib, 19 were identified with JAK2 as the only false negative [35]. HT-CETSA immunoassay and reporter systems have become essential tools for early-stage drug discovery by supporting hit identification, lead optimization, and potency ranking through dose–response analyses. Applications such as B-Raf immunoassay screening and p38α NaLTSA profiling have demonstrated their utility in identifying intracellular target-binding compounds. HT-CETSA provides robust and quantitative data on drug–protein interactions to accelerate the identification and optimization of drug candidates [32]. Table 3 compares the throughputs and applications of HT-CETSA with other CETSA variants.

## 4. Advances in CETSA: From Tissue Applications to Multiomic Integrations

CETSA has significantly advanced drug discovery by enabling the evaluation of target engagement in complex biological environments, including intact tissues and in vivo models. Recent studies have demonstrated its utility in preclinical and clinical applications. For instance, RIPK1 inhibitors were evaluated using Alpha CETSA^®^ and MSD CETSA^®^ formats in unprocessed human whole blood, showcasing high sensitivity and robustness in pharmacokinetic–pharmacodynamic studies [31]. In clinical settings, CETSA has increasingly been adopted to evaluate drug efficacy and safety in human samples. The CETSA^®^ platform developed by Pelago Bioscience has extended its utility to clinical trials, allowing target engagement measurements in unprocessed human blood or tissue biopsies. For example, mass spectrometry-based CETSA formats have enabled the proteome-wide profiling of kinase inhibitors such as Palbociclib and Dinaciclib, providing insights into selectivity and off-target effects [31]. Additionally, CETSA-PISA (parallel integration of sample aliquots) further enables proteome-wide profiling in animal models and patient-derived tissues, facilitating biomarker identification and off-target effect analysis [37]. These developments highlight CETSA’s potential as a clinical diagnostic tool for personalized medicine and patient stratification.

Recent efforts to integrate CETSA with other omics technologies have expanded its utility for understanding drug mechanisms of action. Combining CETSA with phosphoproteomics has revealed dynamic pathway modulations; for example, studies demonstrated how GSK3 inhibition affects PIN1 phosphorylation patterns [38]. These multiomic workflows allow researchers to connect target engagement data with downstream functional outcomes, offering a holistic view of drug activity at the molecular level.

High-throughput CETSA-MS workflows have scaled up significantly, enabling proteome-wide screening across hundreds of compounds. For instance, EUbOPEN’s initiative aims to screen 192 compounds against 1000 targets by 2025 using advanced CETSA-MS pipelines [31]. Computational tools like CycleDNN—a deep learning framework—have emerged to predict CETSA features across cell lines based on existing datasets, reducing experimental costs while expanding applicability. These advancements not only improve scalability but also enhance precision in target identification and validation efforts. As a result, CETSA is becoming a cornerstone technology for multiomic drug discovery and translational research.

## 5. Challenges and Future Improvements

CETSA and its variants have greatly advanced drug discovery and target identification of natural products by providing label-free and physiologically relevant insights into drug–target engagement. However, several limitations must be addressed for their broader application. WB-CETSA is a well-established method for validating drug–target engagement in living cells, but it has the major drawback of relying on antibody-based detection, which restricts its applicability to known proteins with available high-specificity antibodies. This limitation makes it unsuitable for large-scale and unbiased screening of unknown targets of natural products. Furthermore, some proteins exhibit minimal or no detectable shifts in thermal stability upon ligand binding, which reduces the effectiveness of WB-CETSA in identifying certain target proteins. Highly heterogeneous proteins and those that do not undergo clear aggregation upon heat-induced unfolding also present challenges for accurate data interpretation [10].

TPP and MS-CETSA have emerged as a powerful tool for proteome-wide target discovery, yet it faces challenges in detecting low-abundance proteins and distinguishing true targets from artifacts. High-abundance proteins often overshadow low-abundance targets (ex. kinases, transcription factors) in MS-CETSA, leading to false negatives. For instance, MAPK8, a low-abundance kinase, remained undetectable in traditional MS-CETSA but was identified using targeted proximity extension assays (PEA), which improved sensitivity by over 100-fold [39].

A major source of false positives is thermal proximity coaggregation (TPCA), where non-target subunits within a protein complex exhibit shared melting behaviors. For example, methotrexate-treated K562 cells showed TPCA-driven thermal shifts in the chromatin assembly factor-1 (CAF-1) complex due to replication stress rather than direct drug interaction. To mitigate TPCA, statistical frameworks like Slim-TPCA use bootstrapping to filter nonspecific coaggregation, while temperature optimization (ex. three-step protocols) reduces batch effects. Orthogonal validation methods, such as SplitLuc CETSA or PEA, further confirm target engagement without relying solely on thermal shifts [40].

False negatives also arise when natural product–protein interactions fail to induce detectable thermal stability changes. For example, lactate dehydrogenase A (LDHA) inhibitors were missed in MS-CETSA due to low intracellular concentrations but detected via RT-CETSA with ThermLuc reporters [41]. To address this, enrichment strategies (ex. tagged systems like SplitLuc) and targeted MS/PEA enhance sensitivity for low-abundance or stable targets. Replicate designs and dynamic monitoring of TPCA modulation scores further improve reliability [39,42].

Cross-validation with complementary techniques is critical to overcoming CETSA’s limitations. Combining TPP with DARTS or SPROX can validate label-free target discovery, while affinity-based methods (ex. biotin pulldowns) confirm interactions for soluble, validated targets. Integrated workflows, such as CETSA-PEA for kinase profiling or chemical denaturation for stable complexes, have demonstrated improved accuracy. These strategies highlight the importance of multi-method approaches to refine target identification in complex biological environments [43,44,45].

The future of CETSA-based strategies in natural product research lies in expanding their application beyond cell-based models to more physiologically relevant contexts, including in vivo and ex vivo systems. Tissue-CETSA and Tissue-TPP offer significant potential for mapping the target engagement of natural products across multiple organs, which can help researchers study drug distribution, therapeutic efficacy, and potential off-target effects within intact biological systems. These techniques can be particularly valuable for evaluating natural products with complex pharmacokinetics or tissue-specific activities [12,46]. CETSA-based strategies can also be integrated with genetically engineered animal models such as knockout mice to offer a system-level understanding of natural product-induced proteomic alterations and provide indirect evidence of target engagement through global changes in protein expression. Such in vivo studies would not only strengthen preclinical evaluations but also support the optimization of dosing regimens and therapeutic windows in drug development [15]. Other future improvements may include integration with multiomic platforms such as quantitative proteomics, metabolomics, and transcriptomics to facilitate a more comprehensive characterization of the mechanisms of action of natural products by linking target engagement with downstream cellular responses. Improvements in mass spectrometry technologies will further enhance the proteome coverage and sensitivity of CETSA and address the current limitations of detecting low-abundance or membrane-bound target proteins. Clinical applications of CETSA are also emerging as a promising avenue with the potential to monitor target engagement in patient-derived samples including tissues and whole blood, which may pave the way for precision medicine. CETSA and its variants are expected to continue to evolve as indispensable tools for the comprehensive assessment of natural product–target engagement from cellular models to clinical settings and ultimately accelerate the development of therapeutics based on natural products [47].

## Figures and Tables

**Table 1:** Comparative evaluation of CETSA, DARTS, SPROX, and traditional affinity-based methods [8,9].

Method	Sensitivity	Throughput	Application Scope	Advantages	Limitations
CETSA	High (thermal stabilization)	Medium-(Western blot)-to-High (SplitLuc/HTS)	Physiological conditions (intact cells), target engagement, off-target effects, drug resistance analysis	Operates in native cellular environments; detects membrane proteins	Requires protein-specific antibodies for WB; limited to soluble proteins in HTS formats
DARTS	Moderate (protease-dependent)	Low-to-Medium	Cell lysates/purified proteins, novel target discovery, validation of known targets	Label-free; no compound modification; cost-effective	Sensitivity depends on protease choice; challenges with low-abundance targets
SPROX	High (domain-level stability shifts)	Medium-to-High (OnePot 2D)	Lysates, weak binders, domain-specific interaction analysis	Provides binding site information via methionine oxidation	Limited to methionine-containing peptides; requires MS expertise
Affinity-Based	High (if reagents are available)	Low	Purified proteins/lysates, validated target analysis	High specificity; compatible with MS or fluorescence	Requires compound modification (e.g., biotinization); may alter binding properties

**Table 2:** Summary of CETSA applications in natural product research.

Natural Product	Source	Molecular Target	Study Objective	Key Finding
Curcumol [17]	*Curcuma wenyujin*	Nucleolin (NCL)	Validate anticancer mechanism in nasopharyngeal carcinoma	Induces apoptosis by inhibiting NCL-mediated ribosome biogenesis
Matrine [18]	*Sophora flavescens*	Src kinase	Investigate anti-proliferative effects in cancer cells	Inhibits Src phosphorylation, suppressing tumor growth
Rapanone A [19]	*Ardisia japonica*	IMPDH2	Identify anti-neuroinflammatory targets	Selective inhibition of IMPDH2 reduces neuroinflammation
Aurone derivative 1a [20]	Synthetic (inspired by natural aurones)	Vps34 (Class III PI3K)	Screen autophagy modulators	Stabilizes Vps34, enhancing autophagic flux
2′-Hydroxycinnamaldehyde [21]	*Cinnamomum cassia*	STAT3	Uncover STAT3 inhibition in cancer	Direct binding inhibits STAT3 phosphorylation and downstream signaling
Oridonin [22]	*Rabdosia rubescens*	Nucleolin	Study anticancer effects in leukemia	Disrupts nucleolin-RNA interactions, inducing apoptosis
Staurosporine [14]	*Streptomyces staurosporeus*	Pan-kinase (51 kinases)	Profile kinase inhibitor activity	Broad-spectrum kinase inhibition confirmed via proteome-wide thermal shifts
Quinine [23]	Cinchona tree bark	*Plasmodium falciparum* PNP (PfPNP)	Elucidate antimalarial mechanism	Induces conformational changes in PfPNP, blocking purine salvage in malaria
Vioprolide A (VioA) [24]	*Cystobacter violaceus*	Nucleolar protein 14 (NOP14)	Identify anticancer targets in ribosome biogenesis	Disrupts NOP14-NEP1 interaction, halting ribosome assembly
Atractylenolide I [25]	*Atractylodes macrocephala*	PSMD4 (Proteasome subunit)	Study immunomodulatory effects in colorectal cancer	Enhances immunoproteasome activity, boosting antigen presentation and antitumor immunity
Alantolactone [26]	*Inula helenium*	AKR1C1	Investigate anticancer activity in non-small-cell lung cancer	Inhibits AKR1C1, suppressing metastasis and proliferation
Ainsliadimer A (AIN) [27]	*Ainsliaea macrocephala*	PRDX1/PRDX2	Uncover ROS-mediated apoptosis mechanism in colorectal cancer	Covalently binds to PRDX1/2 cysteine residues, inhibiting peroxidase activity and inducing oxidative stress
Capsaicin [28]	*Capsicum annuum*	tNOX (ENOX2)	Validate anticancer mechanism in bladder cancer	Binds tNOX, induces proteasomal degradation, and suppresses SIRT1-mediated G1 cyclin/CDK activation

**Table 3:** Features of CETSA variants.

Method	Detection	Scope	Throughput	Application
WB-CETSA	Western Blot	Target-specific	Low	Target validation
ITDR-CETSA	Western Blot	Target-specific, dose-dependent	Low	Binding affinity assessment
MS-CETSA (TPP)	Mass Spectrometry	Proteome-wide	High	Target discovery
2D-TPP	Mass Spectrometry	Proteome-wide, multidimensional	High	Comprehensive interaction profiling
HT-CETSA	Immunoassay/Reporter System	High-throughput target screening	Very High	High-throughput drug screening and target identification

## References

[1] Newman D.J., Cragg G.M. (2020). Natural Products as Sources of New Drugs over the Nearly Four Decades from 01/1981 to 09/2019. J. Nat. Prod..

[2] Ha J., Park H., Park J., Park S.B. (2021). Recent advances in identifying protein targets in drug discovery. Cell Chem. Biol..

[3] Wang G., Shang L., Burgett A.W., Harran P.G., Wang X. (2007). Diazonamide toxins reveal an unexpected function for ornithine delta-amino transferase in mitotic cell division. Proc. Natl. Acad. Sci. USA.

[4] Harding M.W., Galat A., Uehling D.E., Schreiber S.L. (1989). A receptor for the immunosuppressant FK506 is a cis-trans peptidyl-prolyl isomerase. Nature.

[5] Taunton J., Hassig C.A., Schreiber S.L. (1996). A mammalian histone deacetylase related to the yeast transcriptional regulator Rpd3p. Science.

[6] Sato S., Murata A., Orihara T., Shirakawa T., Suenaga K., Kigoshi H., Uesugi M. (2011). Marine natural product aurilide activates the OPA1-mediated apoptosis by binding to prohibitin. Chem. Biol..

[7] Drahl C., Cravatt B.F., Sorensen E.J. (2005). Protein-reactive natural products. Angew. Chem. Int. Ed. Engl..

[8] Chang J., Kim Y., Kwon H.J. (2016). Advances in identification and validation of protein targets of natural products without chemical modification. Nat. Prod. Rep..

[9] Tolvanen T.A. (2022). Current Advances in CETSA. Front. Mol. Biosci..

[10] Martinez Molina D., Jafari R., Ignatushchenko M., Seki T., Larsson E.A., Dan C., Sreekumar L., Cao Y., Nordlund P. (2013). Monitoring drug target engagement in cells and tissues using the cellular thermal shift assay. Science.

[11] Franken H., Mathieson T., Childs D., Sweetman G.M., Werner T., Tögel I., Doce C., Gade S., Bantscheff M., Drewes G. (2015). Thermal proteome profiling for unbiased identification of direct and indirect drug targets using multiplexed quantitative mass spectrometry. Nat. Protoc..

[12] Martinez Molina D., Nordlund P. (2016). The Cellular Thermal Shift Assay: A Novel Biophysical Assay for In Situ Drug Target Engagement and Mechanistic Biomarker Studies. Annu. Rev. Pharmacol. Toxicol..

[13] Jafari R., Almqvist H., Axelsson H., Ignatushchenko M., Lundbäck T., Nordlund P., Martinez Molina D. (2014). The cellular thermal shift assay for evaluating drug target interactions in cells. Nat. Protoc..

[14] Savitski M.M., Reinhard F.B., Franken H., Werner T., Savitski M.F., Eberhard D., Martinez Molina D., Jafari R., Dovega R.B., Klaeger S. (2014). Tracking cancer drugs in living cells by thermal profiling of the proteome. Science.

[15] Tu Y., Tan L., Tao H., Li Y., Liu H. (2023). CETSA and thermal proteome profiling strategies for target identification and drug discovery of natural products. Phytomedicine.

[16] Dai L., Li Z., Chen D., Jia L., Guo J., Zhao T., Nordlund P. (2020). Target identification and validation of natural products with label-free methodology: A critical review from 2005 to 2020. Pharmacol. Ther..

[17] Wang J., Wu J., Li X., Liu H., Qin J., Bai Z., Chi B., Chen X. (2018). Identification and validation nucleolin as a target of curcumol in nasopharyngeal carcinoma cells. J. Proteom..

[18] Zhang X., Xu H., Bi X., Hou G., Liu A., Zhao Y., Wang G., Cao X. (2021). Src acts as the target of matrine to inhibit the proliferation of cancer cells by regulating phosphorylation signaling pathways. Cell Death Dis..

[19] Liao L.X., Song X.M., Wang L.C., Lv H.N., Chen J.F., Liu D., Fu G., Zhao M.B., Jiang Y., Zeng K.W. (2017). Highly selective inhibition of IMPDH2 provides the basis of antineuroinflammation therapy. Proc. Natl. Acad. Sci. USA.

[20] Li G., Boyle J.W., Ko C.N., Zeng W., Wong V.K.W., Wan J.B., Chan P.W.H., Ma D.L., Leung C.H. (2019). Aurone derivatives as Vps34 inhibitors that modulate autophagy. Acta Pharm. Sin. B.

[21] Yoon Y.J., Kim Y.H., Lee Y.J., Choi J., Kim C.H., Han D.C., Kwon B.M. (2019). 2′-Hydroxycinnamaldehyde inhibits proliferation and induces apoptosis via signal transducer and activator of transcription 3 inactivation and reactive oxygen species generation. Cancer Sci..

[22] Vasaturo M., Cotugno R., Fiengo L., Vinegoni C., Dal Piaz F., De Tommasi N. (2018). The anti-tumor diterpene oridonin is a direct inhibitor of Nucleolin in cancer cells. Sci. Rep..

[23] Dziekan J.M., Yu H., Chen D., Dai L., Wirjanata G., Larsson A., Prabhu N., Sobota R.M., Bozdech Z., Nordlund P. (2019). Identifying purine nucleoside phosphorylase as the target of quinine using cellular thermal shift assay. Sci. Transl. Med..

[24] Kirsch V.C., Orgler C., Braig S., Jeremias I., Auerbach D., Müller R., Vollmar A.M., Sieber S.A. (2020). The Cytotoxic Natural Product Vioprolide A Targets Nucleolar Protein 14, Which Is Essential for Ribosome Biogenesis. Angew. Chem. Int. Ed. Engl..

[25] Xu H., Van der Jeught K., Zhou Z., Zhang L., Yu T., Sun Y., Li Y., Wan C., So K.M., Liu D. (2021). Atractylenolide I enhances responsiveness to immune checkpoint blockade therapy by activating tumor antigen presentation. J. Clin. Investig..

[26] Fu Z., Li S., Liu J., Zhang C., Jian C., Wang L., Zhang Y., Shi C. (2022). Natural Product Alantolactone Targeting AKR1C1 Suppresses Cell Proliferation and Metastasis in Non-Small-Cell Lung Cancer. Front. Pharmacol..

[27] Lv C., Huang Y., Wang Q., Wang C., Hu H., Zhang H., Lu D., Jiang H., Shen R., Zhang W. (2023). Ainsliadimer A induces ROS-mediated apoptosis in colorectal cancer cells via directly targeting peroxiredoxin 1 and 2. Cell Chem. Biol..

[28] Islam A., Su A.J., Zeng Z.M., Chueh P.J., Lin M.H. (2019). Capsaicin Targets tNOX (ENOX2) to Inhibit G1 Cyclin/CDK Complex, as Assessed by the Cellular Thermal Shift Assay (CETSA). Cells.

[29] Almqvist H., Axelsson H., Jafari R., Dan C., Mateus A., Haraldsson M., Larsson A., Martinez Molina D., Artursson P., Lundbäck T. (2016). CETSA screening identifies known and novel thymidylate synthase inhibitors and slow intracellular activation of 5-fluorouracil. Nat. Commun..

[30] Cui Z., Li C., Chen P., Yang H. (2022). An update of label-free protein target identification methods for natural active products. Theranostics.

[31] Wu Q., Zheng J., Sui X., Fu C., Cui X., Liao B., Ji H., Luo Y., He A., Lu X. (2024). High-throughput drug target discovery using a fully automated proteomics sample preparation platform. Chem. Sci..

[32] Henderson M.J., Holbert M.A., Simeonov A., Kallal L.A. (2020). High-Throughput Cellular Thermal Shift Assays in Research and Drug Discovery. SLAS Discov..

[33] Shaw J., Dale I., Hemsley P., Leach L., Dekki N., Orme J.P., Talbot V., Narvaez A.J., Bista M., Martinez Molina D. (2019). Positioning High-Throughput CETSA in Early Drug Discovery through Screening against B-Raf and PARP1. SLAS Discov..

[34] Martinez N.J., Asawa R.R., Cyr M.G., Zakharov A., Urban D.J., Roth J.S., Wallgren E., Klumpp-Thomas C., Coussens N.P., Rai G. (2018). A widely-applicable high-throughput cellular thermal shift assay (CETSA) using split Nano Luciferase. Sci. Rep..

[35] Dart M.L., Machleidt T., Jost E., Schwinn M.K., Robers M.B., Shi C., Kirkland T.A., Killoran M.P., Wilkinson J.M., Hartnett J.R. (2018). Homogeneous Assay for Target Engagement Utilizing Bioluminescent Thermal Shift. ACS Med. Chem. Lett..

[36] McNulty D.E., Bonnette W.G., Qi H., Wang L., Ho T.F., Waszkiewicz A., Kallal L.A., Nagarajan R.P., Stern M., Quinn A.M. (2018). A High-Throughput Dose-Response Cellular Thermal Shift Assay for Rapid Screening of Drug Target Engagement in Living Cells, Exemplified Using SMYD3 and IDO1. SLAS Discov..

[37] Hendricks J.A., Beaton N., Chernobrovkin A., Miele E., Hamza G.M., Ricchiuto P., Tomlinson R.C., Friman T., Borenstain C., Barlaam B. (2022). Mechanistic Insights into a CDK9 Inhibitor Via Orthogonal Proteomics Methods. ACS Chem. Biol..

[38] Lu Q., Zhang Y., Hellner J., Giannini C., Xu X., Pauwels J., Ma Q., Dejonghe W., Han H., Van de Cotte B. (2022). Proteome-wide cellular thermal shift assay reveals unexpected cross-talk between brassinosteroid and auxin signaling. Proc. Natl. Acad. Sci. USA.

[39] Al-Amin R.A., Gallant C.J., Muthelo P.M., Landegren U. (2021). Sensitive Measurement of Drug-Target Engagement by a Cellular Thermal Shift Assay with Multiplex Proximity Extension Readout. Anal. Chem..

[40] Sun S., Zheng Z., Wang J., Li F., He A., Lai K., Zhang S., Lu J.H., Tian R., Tan C.S.H. (2023). Improved in situ characterization of protein complex dynamics at scale with thermal proximity co-aggregation. Nat. Commun..

[41] Sanchez T.W., Ronzetti M.H., Owens A.E., Antony M., Voss T., Wallgren E., Talley D., Balakrishnan K., Leyes Porello S.E., Rai G. (2022). Real-Time Cellular Thermal Shift Assay to Monitor Target Engagement. ACS Chem. Biol..

[42] Sanchez T.W., Owens A., Martinez N.J., Wallgren E., Simeonov A., Henderson M.J. (2021). High-Throughput Detection of Ligand-Protein Binding Using a SplitLuc Cellular Thermal Shift Assay. Methods Mol. Biol..

[43] Friman T. (2020). Mass spectrometry-based Cellular Thermal Shift Assay (CETSA^®^) for target deconvolution in phenotypic drug discovery. Bioorg Med. Chem..

[44] Mateus A., Kurzawa N., Becher I., Sridharan S., Helm D., Stein F., Typas A., Savitski M.M. (2020). Thermal proteome profiling for interrogating protein interactions. Mol. Syst. Biol..

[45] Tan C.S.H., Go K.D., Bisteau X., Dai L., Yong C.H., Prabhu N., Ozturk M.B., Lim Y.T., Sreekumar L., Lengqvist J. (2018). Thermal proximity coaggregation for system-wide profiling of protein complex dynamics in cells. Science.

[46] Mateus A., Kurzawa N., Perrin J., Bergamini G., Savitski M.M. (2022). Drug Target Identification in Tissues by Thermal Proteome Profiling. Annu. Rev. Pharmacol. Toxicol..

[47] Lyu J., Wang K., Ye M. (2020). Modification-free approaches to screen drug targets at proteome level. TrAC Trends Anal. Chem..

